# Recurrent Knee Dislocation With Vascular Injury in a Patient With Prior Total Knee Arthroplasty: A Case Report

**DOI:** 10.7759/cureus.93119

**Published:** 2025-09-24

**Authors:** Taylor J Smith, David Whetstone

**Affiliations:** 1 Emergency Medicine, The University of Tennessee Health Science Center, Nashville, USA

**Keywords:** emergent conditions, knee dislocation, popliteal artery, total knee arthroplasty, vascular injury

## Abstract

Vascular injury after total knee arthroplasty (TKA) is exceedingly rare, with the potential to have catastrophic consequences. This injury has the potential to become more common with the rising incidence of this procedure and an aging population.

We present a case of a 71-year-old woman who suffered from chronic instability and multiple dislocations of TKA following two falls from standing, both of which occurred and were observed in the hospital. She sustained a popliteal artery injury, with a subsequent bypass performed the same day. The course was complicated by recurrent dislocation requiring external fixation, thrombus, infection, and stenosis, all of which required intervention.

There are grave consequences to TKA dislocations, and clinicians should keep a high level of suspicion for and a low threshold to obtain CT angiography in any patient with concern for knee dislocation. TKA incidence will continue to rise as the population ages, and dislocation can lead to significant injury, morbidity, and even mortality. It must be considered, even in low-energy trauma, quickly recognized and treated with specialty assistance.

## Introduction

Traumatic native knee dislocation is a rare phenomenon, accounting for less than 0.5% of all joint dislocations [1]. Tibiofemoral dislocation after a total knee arthroplasty (TKA) is exceedingly rare, reported to be 0.15-0.5% of primary TKAs [2]. Data have suggested that up to 40% of patients with native knee dislocations sustain an associated vascular injury [3]. Estimates of knee prosthesis dislocations with concurrent vascular injury are about 5% [4]. Recurrent dislocation of TKA with a vascular injury further delineates the rarity of our case.

We present the case of a 71-year-old female with a BMI of 40.8, who had a witnessed fall in the emergency department (ED) with obvious knee deformity concerning for dislocation that presumably spontaneously reduced. CT angiography (CTA) revealed no evidence of vascular injury, leading to her discharge with an immobilizer. She sustained another fall, this time with anterior knee dislocation after removing her knee immobilizer with a simultaneous arterial injury requiring an emergent revascularization procedure. Post-operative X-rays revealed recurrent dislocation, which again required reduction with the addition of a knee-spanning external fixator.

A recent case series predicts an increase in the incidence of knee dislocations from falls from height in the context of rising obesity rates [5]. With the most common etiology of TKA dislocation being comorbidity-related (65.2%), with obesity (BMI > 30) the most frequent comorbidity found, this information is vital to be aware of. Given the rarity of TKA-related dislocation and the anticipated significant rise in TKA incidence, we deemed this case distinctive and crucial for emergency medicine physicians, who will undoubtedly need to remain vigilant as the number of TKA patients increases.

## Case presentation

**Figure 1:**
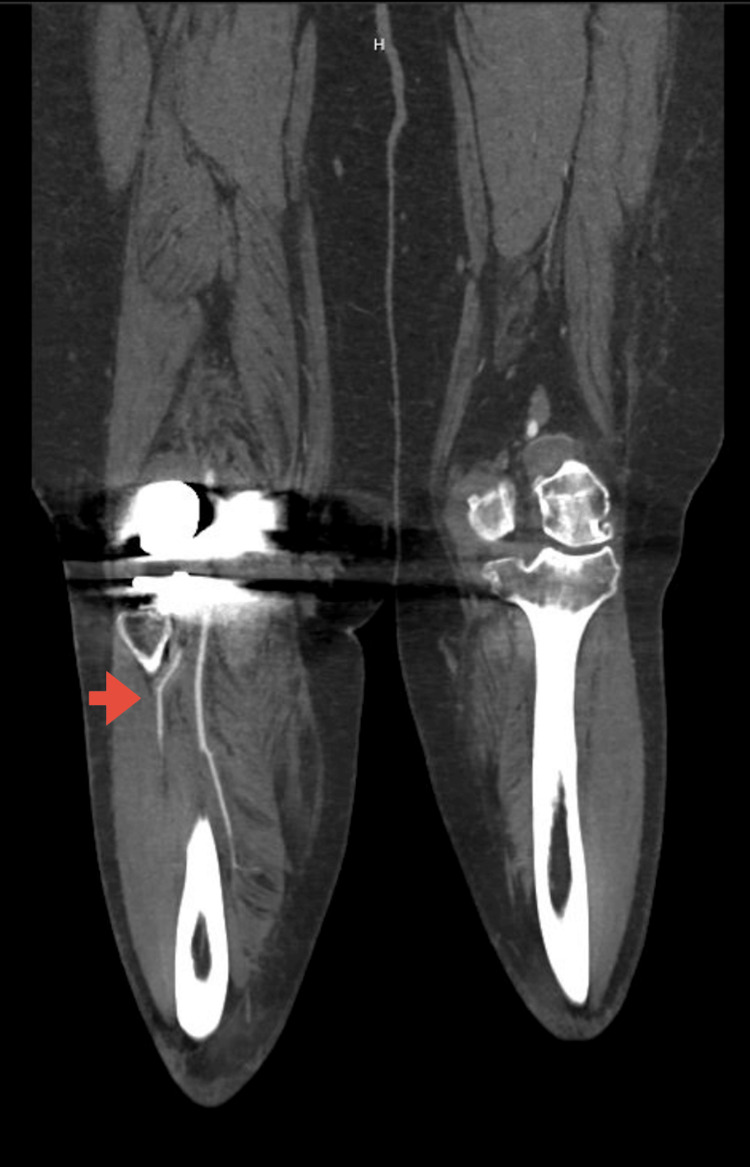
CT angiography (CTA) demonstrating contrast in the runoff vessels (red arrow), indicating the absence of vascular injury.

The patient had a right TKA without complications. Seven months later, the patient had a witnessed fall in the ED, resulting in an obvious lateral deformity to the right lower leg with the patient in a prone position. We assisted the patient into a supine position, and the defect spontaneously resolved, but the joint was felt to be unstable. Further examination showed intact neurovascular status. CTA was performed and showed diffusely patent vasculature with no evidence of arterial disruption, but a streak artifact degraded the P2 and P3 segments of the popliteal artery. There was wide patency of the runoff arteries, and no dislocation was noted (Figure 1).

The patient's pain resolved with intravenous (IV) pain control, and plain films were negative for other pathology in the extremity. She was placed in a knee immobilizer with a normal neurovascular exam. We consulted orthopedics, who recommended discharge with close outpatient follow-up.

**Figure 2:**
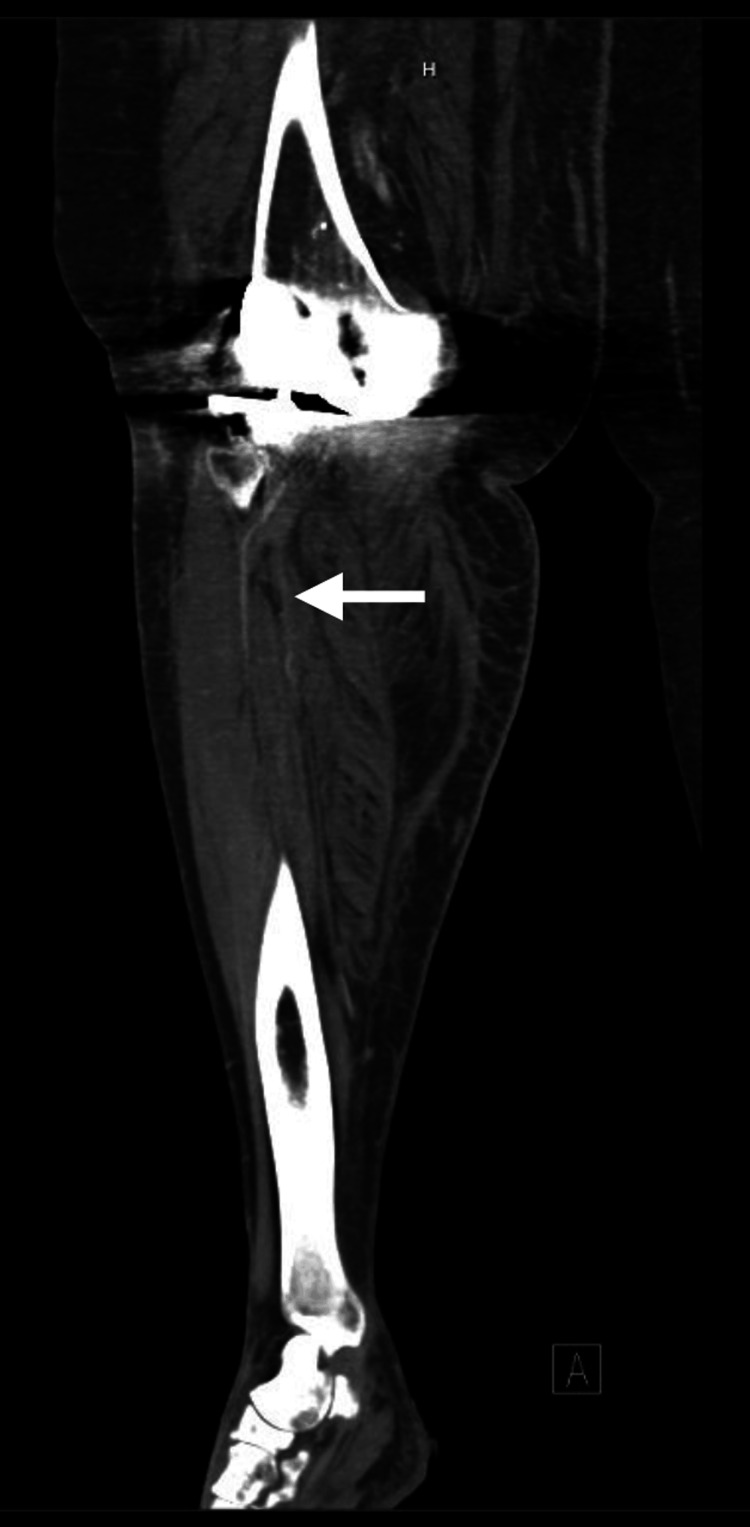
CT angiography (CTA) showing no contrast in the runoff vessels (white arrow), indicating arterial injury.

The patient was staying with her hospitalized daughter and subsequently had another fall two days later when she took her immobilizer off to go to the bathroom. She again had pain, but this time was without a dorsalis pedis or posterior tibialis pulse and had decreased sensation below the knee. Another CTA revealed anterior knee dislocation with evidence of arterial occlusion (Figure 2). The emergency physician astutely discovered a dislocation on the scout film of the CTA and immediately went to the bedside and performed closed reduction with assistance from orthopedic surgery. Vascular surgery took the patient to the operating room (OR) and performed a superficial femoral artery (SFA) to posterior tibial bypass using a reverse saphenous vein graft.

**Figure 3:**
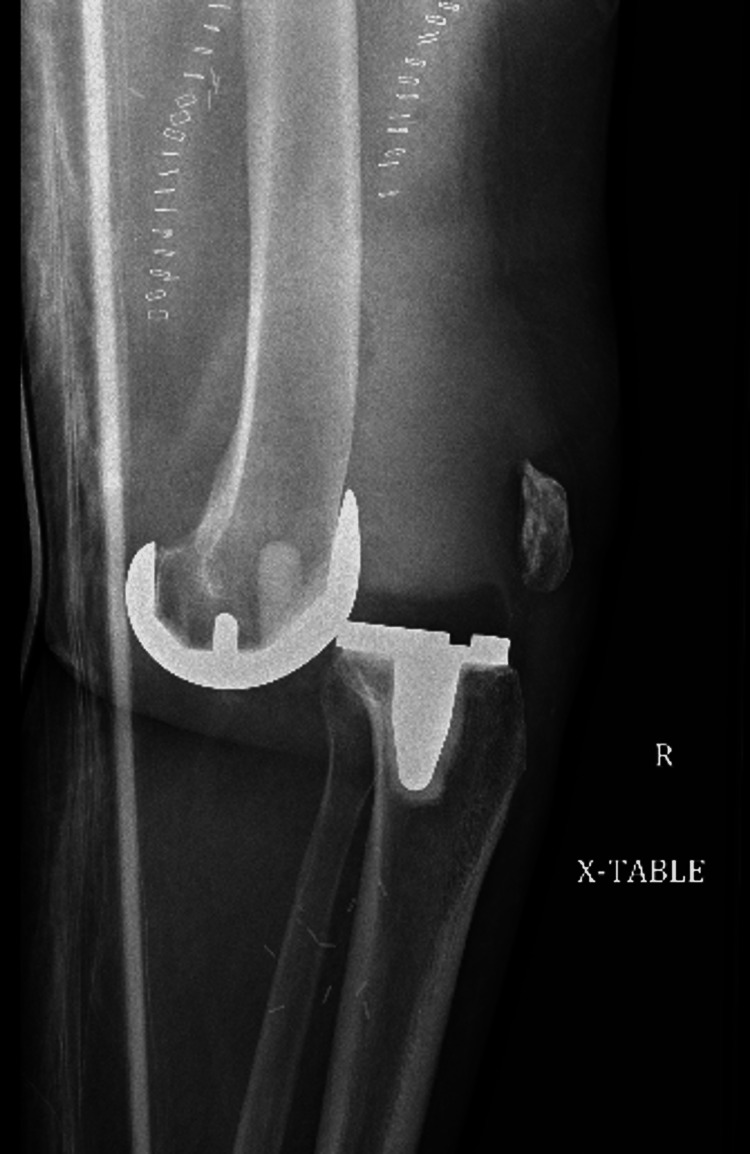
Postoperative day 0 X-ray showing anterior re-dislocation of the joint.

That evening, the patient had repeat X-rays that revealed redislocation of the joint (Figure 3).

She was taken back to the OR the following day for reduction and external fixation by orthopedic surgery, after which she lost pulses, and arterial duplex ultrasound revealed retrograde flow in the runoff vessel, concerning for failure of the bypass or other complication. Emergent consent was obtained from the family while still under general anesthesia for angiogram and re-exploration of the distal bypass anastomosis. A thrombus was discovered at the origin of the posterior tibial artery, which was successfully treated with embolectomy. The patient spent over two weeks in the hospital, complicated by a local skin infection requiring IV antibiotics, and was later transitioned to oral and treated successfully without further intervention. The patient returned six weeks later to have the external fixator removed to check joint stability. It was found stable enough with minimal laxity, and a knee brace was applied with limited extension and flexion.

Three months later, after routine follow-up with vascular surgery, the patient developed abnormal velocities on surveillance ultrasound. She returned to the OR for an angiogram, and it was discovered that she had multifocal 90% stenosis of the proximal posterior tibial distal to the anastomosis, which was successfully treated with balloon angioplasty with 0% residual stenosis. She also had a focal 60% stenosis of the posterior tibial artery proximal to the anastomosis, which was successfully treated with balloon angioplasty, leaving 0% residual stenosis.

## Discussion

Knee dislocation has historically been a rare injury that requires high-energy mechanisms [6]. Low-energy mechanisms are believed to be more common than previously thought, with dislocation in obese individuals being reported more frequently [7-9]. With obesity rates on the rise and more frequent TKAs, we will start to see this rare diagnosis more often. In terms of TKA, epidemiological studies forecast a regular increase of 3-4% in incidence per year up to 2030, but annual cases dropped after COVID-19 and have not yet returned to the previous peak [4,10]. No matter when surgery took place, dislocation must be considered with any trauma, including what presents as simple falls. The mean age at first dislocation was 67.5 ± 10.5 years, and the mean time from primary TKA to first dislocation was 27.1 ± 40.1 months [4]. It can occur at any time after initial surgery; one article presented a case of anterior dislocation with popliteal injury 22 years after the operation [11].

In a systematic review, authors describe three main categories thought to identify the most common etiologies of TKA dislocation: comorbidity-related (65.2%), intraoperative iatrogenic (60.9%), and implant design (12%). They report that obesity (BMI > 30) was the most frequent comorbidity found in 39.2% of patients, followed by severe preoperative deformity, neuropsychiatric disorders (10.1%), and lastly, decompensated metabolic or rheumatologic disease [4].

Associated injuries are common and can include arterial, nerve, fractures, and compartment syndrome. Thorough physical examination is mandatory and should be documented in detail. Nerve injury occurs in 16-40% of dislocations [12], with common peroneal injury occurring in 25-40% of all knee dislocations [13]. The presence of a pulse does not rule out vascular injury; it is only 80% sensitive for detecting a popliteal artery injury [14]. Four percent of patients with normal post-reduction pulses had an arterial injury [15]. Intimal flap tear of the vessel can initially preserve pulses but later result in thrombosis [7]. Timely diagnosis of acute ischemia is imperative due to the catastrophic consequences, including amputation rates as high as 86% if treatment is delayed more than eight hours [16]. Earlier intervention leads to better outcomes. Amputation rates were 6% compared with 11.7% and 13.4% in those undergoing repair within 60 minutes, after one to three hours, and three to six hours, respectively [17].

Treatment for TKA dislocation is initially the same as in a native knee, with immediate and expedited reduction and immobilization in a 15° to 20° flexion semi-rigid splint. Next steps to evaluate for concurrent injury are complex and not necessarily uniformly agreed upon. Native dislocation includes performing the ankle-brachial index (ABI) if there are no hard signs of vascular insufficiency, with the caveat that it can miss intimal flaps/tears and false aneurysms. Every patient with an ABI <0.9 had an arterial injury, and no patient with an ABI ≥0.9 had an arterial injury [18]. After ABI, if appropriate, multiple other diagnostic approaches are used, including admission for serial examinations every two to four hours for 48 hours, angiography for all, and utilization of duplex ultrasound [1,19,20]. Multiple recent articles recommend assessment of the limb’s vascular status with CTA for all patients as soon as possible [21,22].

If vascular injury is discovered, immediate vascular surgical consultation should be obtained, most likely in conjunction with orthopedics, with high rates requiring revision and a possible need for external fixation. One case report recommends that orthopedic surgery be present at the time of bypass surgery to confirm knee reduction and for consideration of external fixation to prevent recurrent dislocation [22]. Recurrence rate for TKA dislocation is common, up to 39.1% (n = 9) when conservative management was chosen, but does report fewer complications, noting eight of these patients ultimately received revision surgery [4]. Based on the limited nature of the current literature for TKA dislocation, to date, there is no validated treatment approach. Some recommend conservative management of splinting for approximately three months and non-weight bearing for four to six weeks [23]. Each case will have to be individualized and will require close communication between multiple surgical specialties.

## Conclusions

We anticipate an increase in a historically rare and difficult-to-diagnose injury. With the rise in morbid obesity and TKAs performed, dislocations from low-energy trauma, including simple falls from height, have the potential to increase the frequency with which we see catastrophic complications of vascular injuries. With pathology as time sensitive as this, emergency medicine and specialist providers must have a higher level of suspicion, and prompt investigation and appropriate management are essential.
